# Supplementary material for: Bacterial-driven development is mediated by Calcium-Dependent Intrinsic Apoptosis in the Squid-Vibrio Symbiosis
Source: bioRxiv. 2026 Jul 16:2026.07.15.738745. Preprint. [Version 1] doi: 10.64898/2026.07.15.738745 (PMC13404735; doi:10.64898/2026.07.15.738745)
Supplement: Supplementary file 1 [file media-4.docx]

**Supplemental figure 1**

PCA of Rlog normalized expression of all appendage RNAseq samples generated in this study. Abbreviations: APO: aposymbiotic, AYM: symbiotic
