# Supplementary material for: Bacterial-driven development is mediated by Calcium-Dependent Intrinsic Apoptosis in the Squid-Vibrio Symbiosis
Source: bioRxiv. 2026 Jul 16:2026.07.15.738745. Preprint. [Version 1] doi: 10.64898/2026.07.15.738745 (PMC13404735; doi:10.64898/2026.07.15.738745)
Supplement: Supplementary file 2 [file media-5.docx]

**Supplemental figure 2**

Negative controls for AIF immunocytochemistry, exposed to only secondary antibody. Nuclei are magenta, secondary antibody signal is in cyan.
