# Supplementary material for: Bacterial-driven development is mediated by Calcium-Dependent Intrinsic Apoptosis in the Squid-Vibrio Symbiosis
Source: bioRxiv. 2026 Jul 16:2026.07.15.738745. Preprint. [Version 1] doi: 10.64898/2026.07.15.738745 (PMC13404735; doi:10.64898/2026.07.15.738745)
Supplement: Supplementary file 3 [file media-6.docx]

**Supplemental figure 3**

Alignment of the epitope region (121-613) for the polyclonal AIF antibody (Invitrogen PA5-48108) and *E. scolopes* AIF
